# Laparoscopic Cholecystectomy for Acute Cholecystitis: Defining the Golden Period

**DOI:** 10.7759/cureus.101426

**Published:** 2026-01-13

**Authors:** Karthik Periyasamy, Aravindan Uthirapthy, M Pon Chidambaram, Senthilkumaran Govindaraj Raman, Sudhagar Rengasamy

**Affiliations:** 1 Surgical Gastroenterology, Thanjavur Medical College, Thanjavur, IND; 2 Surgical Gastroenterology and Gastrointestinal (GI) Oncology, Thanjavur Medical College, Thanjavur, IND; 3 Surgical Gastroenterology and Liver Transplantation, Stanley Medical College, Chennai, IND

**Keywords:** acute cholecystitis, bile leak, intermediate cholecystectomy, laparoscopic cholecystectomy, timing of surgery

## Abstract

Background/aims

Laparoscopic cholecystectomy (LC) performed within the first week of acute cholecystitis (AC) is generally regarded as safe and effective, while delayed surgery is often recommended for unresolved or complicated cases. This study assessed perioperative outcomes of LC performed during the first week versus two to six weeks following symptom onset.

Methods

This prospective observational comparative study was conducted at a tertiary care center from January 2021 to December 2024 using data from a prospectively maintained database. Patients with acute calculous cholecystitis who underwent LC within six weeks of symptom onset were included. Patients with acalculous cholecystitis, choledocholithiasis, spreading peritonitis, or cholecystoenteric/choledochoenteric fistula and emergency open cholecystectomy were excluded. Patients were categorized into Group A (surgery within one week) and Group B (surgery between two and six weeks). Operative duration, conversion to open cholecystectomy, postoperative morbidity, and hospital stay were analyzed.

Results

Among 121 patients, 69 underwent early LC (Group A) and 52 underwent intermediate LC (Group B). Bile leak occurred in 10.1% and 21.2% of cases, respectively (p = 0.057). Mean operative time and subtotal cholecystectomy rates were comparable between groups. The mean duration of hospital stay was approximately 5-6 days (5.68 ± 1.79 days in Group A and 6.21 ± 1.65 days in Group B).

Conclusions

Intermediate LC represents a safe and practical alternative when early surgery is not feasible, provided appropriate patient selection and meticulous surgical technique are ensured. Early LC should remain the treatment of choice whenever feasible, given its well-established benefits of shorter hospital stay, fewer recurrent biliary events, and lower overall costs.

## Introduction

Acute cholecystitis (AC) is one of the most common complications of gallstone disease. Major advances have occurred in the management of AC and complex acute biliary problems in the past few years. These changes include earlier surgery and index admission cholecystectomy [1,2]. As surgical techniques improved, laparoscopic cholecystectomy (LC) became widely accepted as the standard of care, even in the emergency setting, offering reduced pain, shorter recovery, and fewer wound-related complications compared with open cholecystectomy.

Initially, surgery was recommended within 72 hours of symptom onset, a period termed the “golden period.” However, the precise definition of this period has varied across studies. Early literature supported surgery within 72 hours, citing lower conversion rates and reduced complications [3]. More recent evidence and updated Tokyo Guidelines 2018 (TG18) [4] and World Society of Emergency Surgery (WSES) 2016 guidelines [5] have extended this safe window to up to 7-10 days after symptom onset. Therefore, in this study, the term “golden period” refers to the first week following symptom onset, aligning with contemporary international recommendations [4,5]. Operations performed beyond the first week have been considered technically more demanding due to dense adhesions and higher conversion rates, leading many surgeons to postpone intervention until six weeks or later [6-8]. During the intervening second to sixth week, cholecystectomy is typically undertaken only for unresolved inflammation.

Despite these concerns, emerging evidence suggests that LC can be performed safely in both early and intermediate phases, provided the surgical team has sufficient experience. Our institution frequently receives referrals of patients beyond the first week of illness. Hence, we conducted this study to compare the results of LC performed during the first week versus two to six weeks from the onset of AC. In this study, we hypothesize that LC performed between two and six weeks would have comparable perioperative outcomes to early surgery in terms of conversion, morbidity, and mortality.

## Materials and methods

Objective

The objective of the study is to compare the operative and postoperative outcomes of early (≤7 days) versus intermediate (2-6 weeks) LC in patients with acute calculous cholecystitis.

Primary outcomes

Primary outcomes include incidence of bile leak, conversion to open cholecystectomy, bile duct injury, and requirement for subtotal cholecystectomy.

Secondary outcomes

Secondary outcomes include operative time (in minutes), postoperative hospital stay (in days), rate of drain placement, and incidence of minor complications (such as wound infection or transient postoperative fever).

Study design and setting

This was a single-center, prospective observational comparative study conducted in the Department of Surgical Gastroenterology & GI Oncology, Thanjavur Medical College and Hospital, Tamil Nadu, India. Data were collected prospectively for all patients admitted with a diagnosis of AC between January 2021 and December 2024 who subsequently underwent LC within six weeks of symptom onset. Institutional Ethics Committee approval was obtained (No. 1329/2024).

Patient selection

Patients were allocated into two groups based on the timing of surgery: Group A: LC within seven days of onset (early cholecystectomy) and Group B: LC between two and six weeks (intermediate cholecystectomy). No formal sample size calculation was performed prior to the study, as all consecutive eligible patients during the study period were included. Inclusion criteria were patients ≥ 18 years with AC (including complicated forms). Exclusion criteria were acute acalculous cholecystitis, AC with choledocholithiasis, generalized peritonitis, or cholecystoenteric fistula and emergency open cholecystectomy.

Diagnosis and preoperative assessment

All patients underwent uniform preoperative optimization and perioperative management as per departmental protocol, including disease severity assessment based on the TG18 classification, which grades AC as mild (Grade I), moderate (Grade II), or severe (Grade III) according to clinical and organ dysfunction criteria [4], and performance of CT for suspected perforation/gangrene and magnetic resonance cholangiopancreatography (MRCP) for suspected common bile duct (CBD) stones. Risk stratification employed the Charlson Comorbidity Index (CCI) [9] and the American Society of Anesthesiologists (ASA) physical status [10] and antibiotic prophylaxis (third-generation cephalosporin with metronidazole), thromboprophylaxis, and postoperative analgesia.

Surgical technique

The consistently low conversion and complication rates in our series likely reflect the surgical team, which comprised three certified consultant surgical gastroenterologists, each with over 10 years of experience in advanced laparoscopic and hepatobiliary procedures and more than 500 prior laparoscopic cholecystectomies performed individually, at our tertiary center. All patients underwent LC under general anesthesia using a standard four-port approach. When necessary, an additional 5 mm port was used for retraction. Dissection of Calot’s triangle was attempted to obtain the critical view of safety (CVS) [11]. In cases where safe dissection was not possible, when the CVS could not be achieved due to dense adhesions, severe inflammation, bleeding obscuring anatomy, or non-identification of the cystic duct and artery despite meticulous dissection, a subtotal cholecystectomy (either reconstituting or fenestrating type) or conversion to open surgery was performed to ensure patient safety.

Although a formal intraoperative difficulty scoring system was not prospectively applied, the assessment was made using criteria similar to those described by Nassar et al. [12]. The gallbladder was retrieved in an endobag via the umbilical port. Drains were placed selectively and removed within 48 hours unless bile leakage was suspected.

Statistical analysis was performed using descriptive and comparative methods (Chi-squared test, t-test) to identify group differences. Multivariate logistic regression was considered but not performed due to the limited sample size and small number of outcome events, which would reduce model reliability. A p-value < 0.05 was significant.

## Results

**Figure 1:**
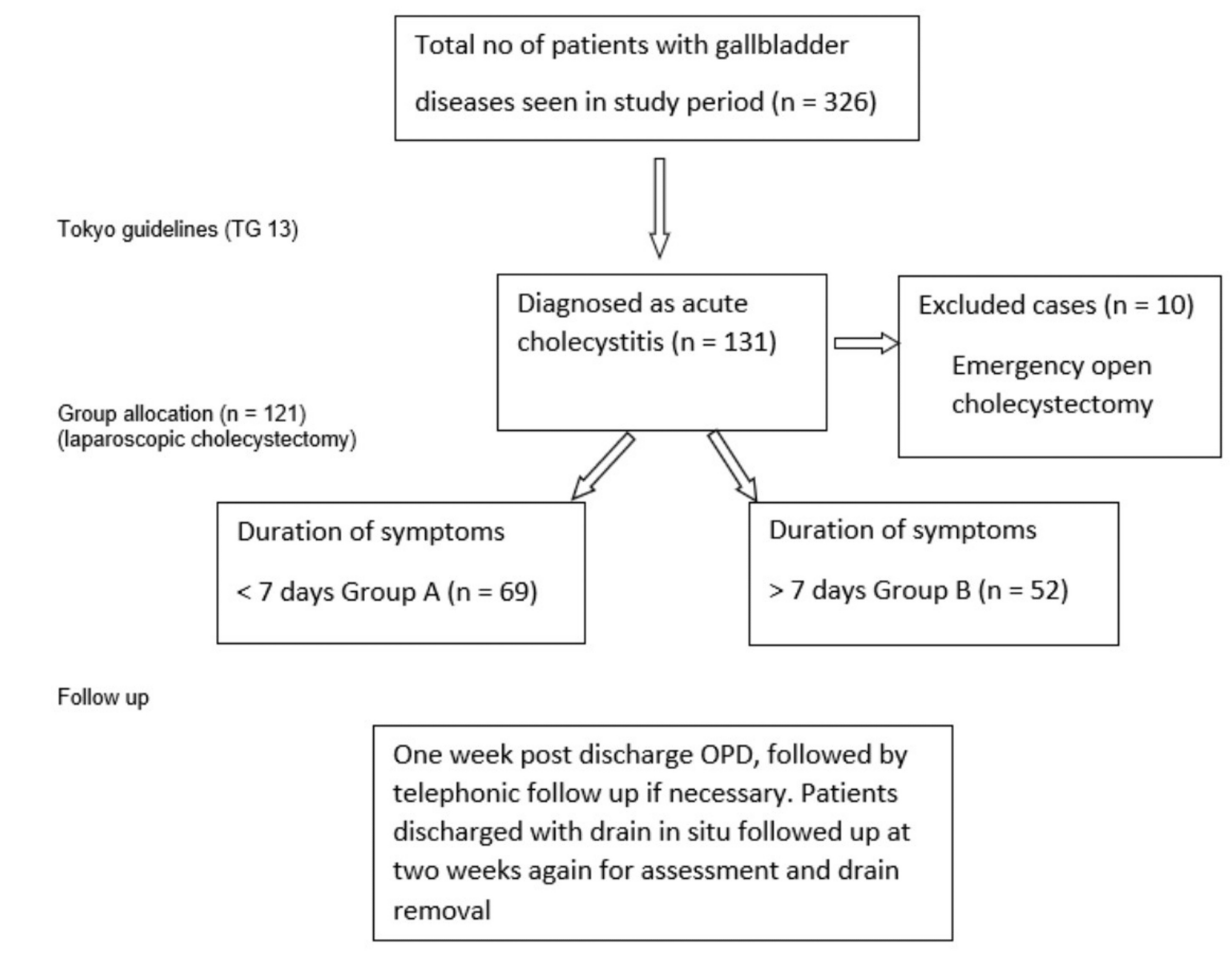
Flowchart showing the selection of study subjects Open cholecystectomy cases (n = 10) were excluded due to the unavailability of an emergency laparoscopic setup.

Of 326 patients with gallstone disease, 131 were diagnosed with AC. After exclusions, 121 underwent LC and were analyzed (Group A: n = 69, Group B: n = 52) (Figure 1).

Baseline demographic characteristics, including age and gender distribution, were comparable between the early and intermediate groups (Table 1).

**Table 1:** Demographics and clinical characteristics

Demography	Duration	
	Group A (1 week)	Group B (2-6 weeks)
Males	33 (47.8%)	24 (46.2%)
			Females	36 (52.2%)	28 (53.8%)
Age (years)	51.45 (±16.07)	49.5 (±15.2)			

Among clinical presentations, pain was the predominant symptom, often accompanied by fever and a positive Murphy’s sign. Laboratory parameters were comparable between the two groups. Diabetes was the most common associated comorbidity. Liver function test abnormalities were infrequent and mild, with comparable rates of bilirubin and transaminase elevation between groups. The distribution of comorbidities (mainly diabetes and hypertension) and clinical diagnoses such as empyema, gangrenous, or perforated gallbladder were also similar, with no statistically significant association (Table 2).

**Table 2:** Clinical presentation, lab reports, and final diagnosis Values are presented as number (%) or mean (range). No statistical significance at p > 0.05 level. Statistical significance at p < 0.05 level. p-value calculated by the Chi-squared test. SGOT: serum glutamic oxaloacetic transaminase; SGPT: serum glutamic pyruvic transaminase

	Duration		p-value
	Group A (1 week)	Group B (2-6 weeks)	
	(n = 69)	(n = 52)	
Clinical presentation			
Pain	68 (98.6%)	52 (100%)	0.57
				Fever	55 (79.7%)	38 (73.1%)	0.323
Murphy’s sign	31 (44.9%)	18 (34.6%)	0.253				
				No. of episodes			
0	54 (78.3%)	35 (67.3%)	0.052
				1	14 (20.2%)	15 (28.84%)
2	1 (1.5%)	2 (3.84%)				
				Hemogram			
Leukocytosis	38 (55.1%)	28 (53.8%)		0.893
					Liver function test			
Total bilirubin	2 (2.9%)	0 (0%)	0.323
				SGOT	1 (1.4%)	1 (1.9%)	0.494
SGPT	1 (1.4%)	1 (1.9%)	0.494				
				Comorbidities			
Diabetes	21 (30.4%)	16 (30.8%)	0.968
				Hypertension	4 (5.8%)	3 (5.8%)	0.582
Other	12 (17.4%)	10 (19.2%)	0.795				
				Clinical diagnosis			
Empyema gallbladder	30 (43.5%)	26 (50%)	0.476
				Gangrene/perforation of gallbladder	12 (17.4%)	8 (15.4%)	0.769
Acute calculus cholecystitis	61 (88.4%)	47 (90.4%)	0.228				
				Acute cholecystitis with pancreatitis	10 (14.5%)	5 (9.6%)	0.42

Intraoperative assessment revealed thickened and inflamed gallbladder walls in more than 95% of the patients in both groups. The incidence of gangrenous or perforated gallbladders and stones impacted at Hartmann’s pouch was observed more frequently in the intermediate group. Conversion to open surgery was uncommon overall and limited to a few early cases (4.3%) (Table 3).

**Table 3:** Operative details Values are presented as number (%) or mean (range). No statistical significance at p > 0.05 level. p-value calculated by Chi-squared test.

Operative details	Duration		p-value
	Group A (1 week)	Group B (2-6 weeks)	
	(n = 69)	(n = 52)	
Thick-walled, edematous, inflamed gallbladder	67 (97.1%)	51 (98.1%)	0.424
				Gangrene/perforation of gallbladder	12 (17.4%)	11 (21.2%)	0.602
Impacted stone in Hartmann’s pouch	47 (68.1%)	39 (75%)	0.408				
				Both cystic duct & artery identifiable	60 (87%)	43 (82.7%)	0.514
Subtotal cholecystectomy	10 (14.5%)	12 (23.1%)	0.226				
				Drain placement	32 (46.4%)	35 (67.3%)	0.022
Conversion to open cholecystectomy	3 (4.3%)	0 (0%)	0.182				
				Duration of surgery (min)			0.104
<60 min	19 (27.53%)	8 (15.38%)					
			60-120 min	32 (46.37%)	34 (65.38%)		
>120 min	18 (26.08%)	10 (19.23%)					
			Mean duration of hospital stay (days)	5.68 (±1.79)	6.21 (±1.65)	0.098	
Postoperative complications							
Bile leak	7 (10.1%)	11 (21.2%)	0.057				
				Others	2 (2.9%)	3 (5.8%)	0.261

Postoperative outcomes

Operative duration was comparable between groups, most often requiring less than two hours, while the need for prolonged duration surgery was found to be more in Group A. Group B showed higher bile leak and drain placement rates and a greater need for subtotal cholecystectomy, indicating increased technical difficulty. Minor complications such as fever and wound infection were infrequent (<6%) (Table 3). The mean postoperative stay was similar in both groups, averaging around six days (Table 3).

## Discussion

In the early laparoscopic era, LC for acute calculous cholecystitis (AC) was considered unsafe because of distorted anatomy and concern for bile duct injury, and only a minority of surgeons attempted it [13,14]. With accumulated experience and improved technique, LC has now emerged as the standard treatment for AC, being routinely performed by more than 90% of surgeons worldwide [15].

The bile leak rate was higher in the intermediate group (21.2% vs. 10.1%), exceeding the 0.3%-2.7% reported internationally [16,17]. However, the difference in bile leak rates (p = 0.057) did not reach statistical significance. The higher incidence is more likely attributed to greater technical difficulty and a higher proportion of subtotal cholecystectomies. The above patients underwent MRCP at the end of the second postoperative week to exclude major bile duct injury or other pathology. The majority of leaks were low-output (<100 mL/day), resolved with conservative management within 10-14 days, and did not require endoscopic retrograde cholangiopancreatography (ERCP), stenting, or reoperation.

The rate of subtotal cholecystectomy in our cohort was 14.5% in Group A and 23.1% in Group B, higher than the 5%-10% reported in most contemporary series [12,16,17]. This higher rate likely reflects the complex nature of cases referred to our tertiary care center, where many patients presented after one week with advanced inflammation, empyema, or gangrenous gallbladders. Previous studies have similarly shown that subtotal cholecystectomy is often required in technically difficult cases to prevent bile duct injury and to ensure safe completion [6,7].

Our conversion rate (4.3% in the early group, 0% in the intermediate group) was favorable compared with international reports of 6%-34% [14,15,18,19]. The low rate likely reflects the experience of the surgical team, adherence to the CVS, and prompt conversion when safe dissection could not be achieved. Also, drain use was significantly higher in Group B (67.3% vs. 46.4%, p = 0.013), as reported in studies from other tertiary centers [6] managing delayed or complicated cholecystitis, where selective drain use aids early detection of bile leaks and safe postoperative management.

The mean hospital stay of approximately six days was similar to the study reported by Gutt et al. [6]. This may be owing to institutional protocols favoring routine drain placement and observation until clinical and biochemical recovery, particularly for patients undergoing subtotal cholecystectomies.

The mean operative duration in our study ranged between 60 and 120 minutes in most cases, with no significant difference between the early and intermediate groups. Slightly longer procedures were observed in the early group, likely reflecting increased vascular congestion and edema during the acute inflammatory phase. Similar observations have been reported in previous studies, where early cholecystectomy often required more meticulous dissection due to friable tissues and oozing, while intermediate cases were technically challenging because of fibrosis and adhesions [6,7].

Minor postoperative complications include wound infections and postoperative fever, which were infrequent and comparable between the two groups and consistent with previous studies [8]. During the one-year follow-up, no patient developed late biliary complications or recurrent symptoms, indicating durable outcomes in both groups.

Although the overall outcomes were acceptable in both groups, the intermediate group demonstrated a higher bile leak rate, greater drain usage, and more frequent subtotal cholecystectomy, all of which indicate increased technical difficulty. These findings suggest that while intermediate LC can be performed feasibly in experienced hands, it does not carry the same ease or risk profile as early surgery.

The strengths of our study include prospective data collection, a homogenous single-center surgical team, and procedures performed under the supervision of experienced hepatobiliary surgeons, which minimized inter-operator variability. The study has certain limitations, including a modest sample size, the absence of a formal intraoperative difficulty grading system, not including a tabulated comparison of TG18 severity grades, and a lack of long-term follow-up to assess recurrent symptoms or late biliary complications. A potential selection bias exists, as patients were not randomized; allocation to early or intermediate surgery was determined by time of presentation and clinical suitability rather than random assignment. Although the trends observed, particularly in bile leak rates, were clinically meaningful, the available sample may have been insufficient to demonstrate statistical significance. Multivariate logistic regression to adjust for confounders such as disease severity, diabetes, previous cholecystitis episodes, age, and comorbidities could not be conducted due to limited event numbers.

## Conclusions

Early LC should remain the preferred approach whenever feasible because of its proven advantages-shorter hospitalization, fewer recurrent attacks, and reduced cost. Intermediate LC serves as a practical alternative when early surgery is not possible, provided that careful patient selection and meticulous surgical technique are observed. Further large-scale, multicenter prospective studies are warranted to validate these findings and refine guidelines for optimal timing of surgery.
